# Effects of Water Quality Adjusted by Submerged Macrophytes on the Richness of the Epiphytic Algal Community

**DOI:** 10.3389/fpls.2018.01980

**Published:** 2019-01-09

**Authors:** Tian Lv, Qiankun He, Yaping Hong, Chunhua Liu, Dan Yu

**Affiliations:** The National Field Station of Freshwater Ecosystem of Liangzi Lake, College of Life Sciences, Wuhan University, Wuhan, China

**Keywords:** submerged macrophytes, coverage, epiphytic algae, richness, water quality

## Abstract

Submerged macrophytes and epiphytic algae play significant roles in the functioning of aquatic ecosystems. Submerged macrophytes can influence the epiphytic algal community by directly or indirectly modifying environmental conditions (nutrients, light, etc.). From December to June of the following year, we investigated the dynamics of the dominant winter species *Potamogeton crispus*, its epiphytic algae, and water quality parameters in the shallow Liangzi Lake in China. The richness of epiphytic algae had a trend similar to that of *P. crispus* coverage, which increased in the first four months and then decreased in the following three months. The structural equation model (SEM) showed that *P. crispus* affected the richness of epiphytic algae by reducing nutrient concentrations (reduction in total organic carbon, total nitrogen and chemical oxygen demand) and enhancing water transparency (reduction in turbidity and total suspend solids) to enhance the richness of epiphytic algae. The results indicated that high amounts of submerged macrophyte cover can increase the richness of the epiphytic algal community by changing water quality.

## Introduction

A least-disturbed shallow ecosystem should have high water quality and biodiversity (Mcnaughton, 1988; Downing et al., 2014). Submerged macrophytes play a significant role in maintaining good water quality and high biodiversity in shallow ecosystems (Jeppesen et al., 1998; Kuiper et al., 2017). Submerged macrophytes inhibit algal blooms through the reduction of nutrients, allelopathy and shading (Nakai et al., 2000; Engelhardt and Ritchie, 2001; Casartelli and Ferragut, 2018). Epiphytic algae play a significant role in the functioning of shallow ecosystems, contributing to material circulation, energy flow and the maintenance of food webs (Rodusky et al., 2001; Vadeboncoeur and Steinman, 2002; Song et al., 2017). The community structure of epiphytic algae in shallow ecosystems is influenced by a number of physical (Vadeboncoeur et al., 2003; Tóth and Palmer, 2016), chemical (Rodusky et al., 2001; Trochine et al., 2014) and biological factors (Jones and Sayer, 2003; Tunca et al., 2014; Hao et al., 2017).

It is widely acknowledged that the relationship between epiphytic algae and macrophytes plays an important role in maintaining the function and stability of the shallow ecosystems (Liboriussen and Jeppesen, 2006; Scheffer and Nes, 2007). The epiphytic algal community can be strongly influenced by macrophytes, especially with a high coverage of macrophytes, which has been frequently reported by researchers (Chambers et al., 2008; Santos et al., 2013; Souza et al., 2015). The macrophytes can directly or indirectly modify the environmental conditions for epiphytic algae, which complicates the relationship between them. Macrophytes participate in the nutrient cycling process through nutrition absorption, precipitation, mobilization, decomposition (Carignan and Kalff, 1980; Barko and James, 1998), processes that can change nutrient and light conditions for epiphytic algae. Macrophytes can provide surfaces for epiphytic algae development, but they may also decrease epiphytic algae growth through reduced light availability due to shading and allelochemical production (Erhard and Gross, 2006; Meerhoff et al., 2007). Therefore, it is reasonable to assume that macrophytes may be a determinant of the community structure of epiphytic algae.

However, few studies have identified a model for variation in the epiphytic algal community, especially considering the effects of water quality changes by aquatic macrophytes on the epiphytic algal community. Therefore, we surveyed the interrelationship between the epiphytic algal community, macrophyte coverage and water quality variables to determine the direct and indirect effects on the epiphytic algal community.

## Materials and Methods

### Study Area

**FIGURE 1:**
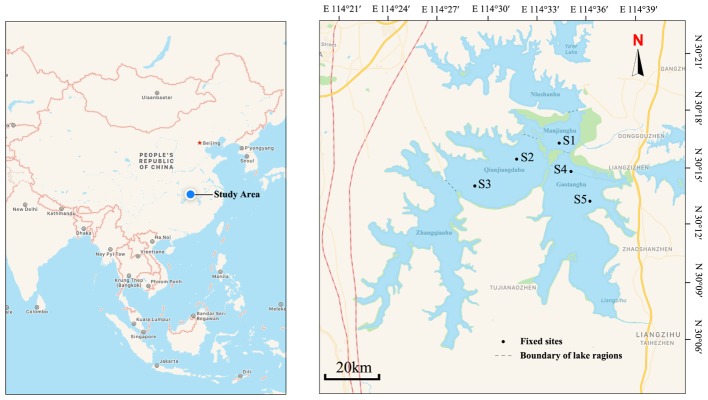
Map of Liangzi Lake showing sampling sites. Five fixed sites were distributed in three lake regions: S1 in Manjianghu, S2 and S3 in Qianjiangdahu, S4 and S5 in Gaotanghu.

Liangzi Lake, Hubei Province, China (30°05′ ∼ 30°18′N, 114°21′ ∼ 114°39′E) is a typical Macrophyte-dominated mesotrophic shallow lake [average annual diaphaneity is 1.2 m, average annual pH is 8.0, average annual salinity is 0.07 ppt, average annual total suspended solids is 19.0 mg/L, average annual total nitrogen is 0.53 mg/L, average annual total phosphorus is 0.023 mg/L and average annual chemical oxygen demand (COD) is 3.68 mg/L] in the central of Yangtze River Basin with good water quality and high biodiversity (Xie et al., 2015). It has a surface area of 304.3 km^2^ and the mean depth varies from 2.5 to 6 m (Fan et al., 2015). It is a dimictic lake, water retention is 0.53 year, about 1.48 × 10^9^ m^3^ water take part in the water replacement each year because of seasonal precipitation and draining into the Yangtze river. Liangzi Lake features a subtropical monsoon climate, and the weather is relatively moderate with an annual average temperature of 17.3°C, the mean freezing period is 15 days. This lake was divided into five regions based on different macrophyte community composition and hydrologic conditions (Figure 1) (Xu et al., 2018). *P. crispus* is an annual submerged macrophyte and a dominant winter species in Liangzi Lake (Qian et al., 2014). It germinated in the autumn (September to November) and slowly grew throughout the winter (December to the coming February, there is no ice, average temp is 7.9°C), increased its biomass rapidly from March to April and declined in June (Rogers and Breen, 1980; Kunii, 1982; Chen, 1985).

### *P. crispus* and Epiphytic Algae Samples

Due to enclosure of other two regions, five fixed sampling sites were distributed in three regions of Liangzi lake (Qianjiangdahu, Manjianghu, and Gaotanghu) (Figure 1). From December 2016 to June 2017, each site was surveyed on the 15th–17th day each month (total of seven times samples). Five quadrats (1 m × 1 m, quadrats were placed without overlapping, randomly) with a *P. crispus* monodominant community were investigated at each site, and the plant coverage of each sample was surveyed by ocular estimate (Fang et al., 2009). The coverage of *P. crispus* (macrophyte cover) at each site was calculated as the mean of the five samples. Ten pieces of *P. crispus* leaves approximately 50 cm from the top were carefully selected from those five quadrats to ensure uniformity in the growth state (young leaf) and size to ensure the minimum sampling error in sample size. Then, each leaf was placed into a wide-mouth plastic bottle with 200 ml of distilled water at each site. The area of the selected leaves was measured by area meter (LI-3100C, LI-COR, United States). Epiphytic algae were removed by a banister brush in water (Foerster and Schlichting, 1965) and preserved in a well-labeled plastic container, with 2 ml Lugol’s solution to fix the epiphytic algal sample. Epiphytic algae were identified to species and quantified with a microscope using the blood count plate method (Hu and Wei, 2006; Effiong and Inyang, 2015; Qian et al., 2015). The total abundance of each month was the mean of the five fixed sites. The richness of epiphytic algae was the summation of species at each site per month.

### Water Samples

Eighteen physical and chemical water parameters were measured at a depth of 1.5 m underwater. Water temperature (T), dissolved oxygen (DO), conductivity (Cond) and pH of water samples were measured with a portable water quality monitor (PROPLUS, YSI, United States), and chlorophyll a (Chla) was measured with a handheld probe (HYDROLAB DS5, HACH, United States). Turbidity (Turb) and total suspended solids (SS) were measured with a turbidity meter (2100Q, HACH, United States) and a portable spectrophotometer (DR900, HACH, United States) in the field tests. Additionally, water samples were collected from each site and stored on ice. Total nitrogen (TN) and total phosphorus (TP) were analyzed by a flow injection analyzer (QC8500, LACHAT, United States), total organic carbon (TOC) was analyzed by a total organic carbon analyzer (TOC-L, SHIMADZU, Japan), the cations and anions (Na^+^, K^+^, Mg^2+^, Ca^2+^, F^-^, Cl^-^, and SO_4_^2-^) were determined by a ion chromatograph (ICS-1000, DIONEX, United States) and COD was analyzed with a digestion solution for COD and landscape photometry (DR900, HACH, United States).

### Data Analyses

To ensure that the data conform to a normal distribution, all water parameters were log_10_-transformed before performing regressions and SEM (Zuur et al., 2010), whereas macrophyte cover and epiphytic richness were not log_10_-transformed (O’Hara and Kotze, 2010). Macrophyte cover and epiphytic algal richness in different months were compared using repeated-measures ANOVA by *post hoc* Bonferroni tests for multiple comparisons (Thompson et al., 2001). The linear regressions were used to test the patterns of epiphytic algal richness along significant environmental gradients and the regressions coefficients squared were corrected for multiple tests. To determine the relative importance of direct vs. indirect effects of *P. crispus* community dynamics driving epiphytic algal richness, we built a structural equation model (SEM; Oberski et al., 2014) including macrophyte cover, nutrient environmental factors (i.e., TN, TP, COD, and TOC), and light-related environmental factors (i.e., Turb and SS), with richness of epiphytic algae. Statistics were performed using R version 3.5.1 (R Development Core Team, 2011) and the packages agricolae (Mendiburu, 2009) and lavaan (Rosseel et al., 2011).

## Results

### Physical and Chemical Parameters

**FIGURE 2:**
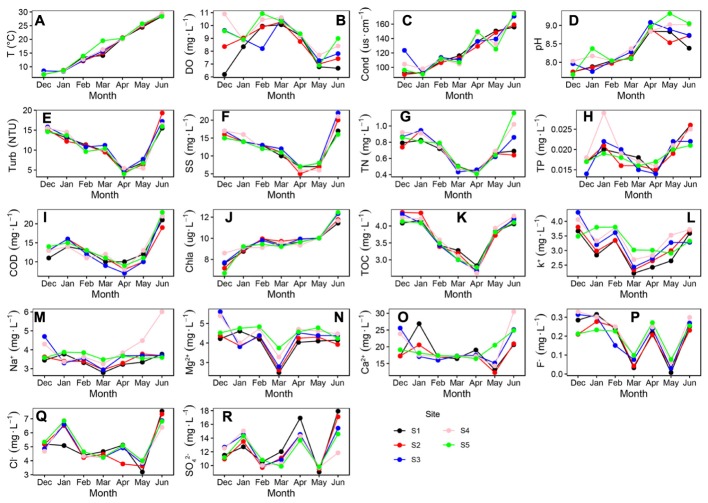
Variations in some physical and chemical parameters of Liangzi Lake (December 2016–June 2017). **(A)** water temperature, **(B)** dissolved oxygen, **(C)** conductivity, **(D)** pH, **(E)** Turbidity, **(F)** total suspended solids, **(G)** Total nitrogen, **(H)** total phosphorus, **(I)** chemical oxygen demand (COD), **(J)** chlorophyll a, **(K)** total organic carbon, **(L)** K^+^ **(M)** Na^+^, **(N)** Mg^2+^, **(O)** Ca^2+^, **(P)** F^-^, **(Q)** Cl^-^ and **(R)** SO_4_^2-^ (*n* = 35).

The T (*P* < 0.001), Cond (*P* < 0.001), pH (*P* < 0.001) and Chla (*P* < 0.001) showed an increasing trend during the survey periods (Figures 2A,C,D,J). The DO Turb, SS, TN, TP, TOC, COD, Na^+^, K^+^, Mg^2+^, Ca^2+^, F^-^, Cl^-^, and SO_4_^2-^ were shown a non-liner trend during the survey periods (Figures 2B,E–I,K–R). Turb, SS, TN, TP, TOC, COD decreased in the first four months of the observation period, followed by increases in the remaining observation period (Figures 2E–I,K). The six values (i.e., Turb, SS, TN, TP, TOC, and COD) in April were smaller than those in other months, which indicated that the water column was cleaner in April than in other months.

### Coverage of *P. crispus* and Epiphytic Algae

**FIGURE 3:**
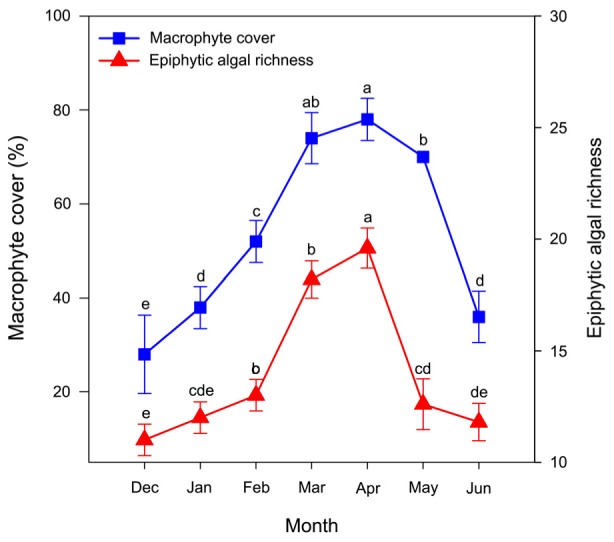
Community dynamics of *P. crispus* and epiphytic algae. The data are presented as the mean ± SE of 5 fixed sites each month (*n* = 35). Means with the different letters are significantly different at *P* < 0.05 in different months (Bonferroni test).

Macrophyte cover (*F*_(2.07,8.29)_ = 72.67, *P* < 0.001) and epiphytic algal richness (*F*_(2.60,10.40)_ = 96.53, *P* < 0.001) were significantly difference in month. macrophyte cover increased in the first four months and then decreased in the last three months (Figure 3). macrophyte cover reached a peak of approximately 75–80% in mid-April (Figure 3). The mean macrophyte cover increased from 28% in December to 38% in January of following the year, showing that *P. crispus* slowly grew throughout the winter. The mean macrophyte cover increased from 38% in January to 78% in April, showing that *P. crispus* increased its biomass rapidly in the spring (Figure 3). The mean macrophyte cover decreased from 78% in April to 36% in June, showing that *P. crispus* declined in the early summer (Figure 3).

**FIGURE 4:**
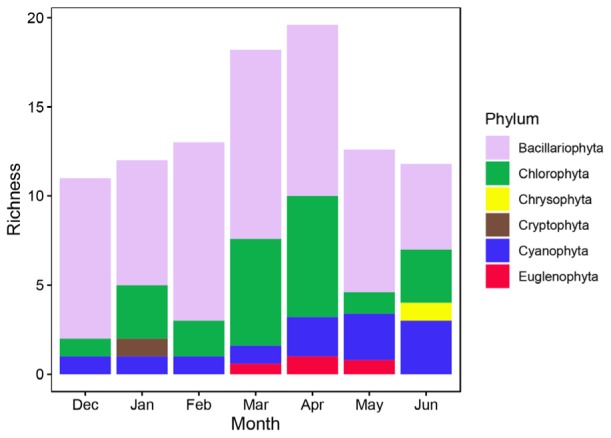
The richness of 6 phyla of epiphytic algae on *P. crispus* during the study period. The richness of each phylum was the mean of 5 fixed sites (*n* = 35).

The richness of epiphytic algae had a trend similar to that of *P. crispus* coverage dynamics, first increasing during the first four months and then decreasing during the last three months (Figure 3). The richness of epiphytic algae reached a peak at approximately 20 species in the mid-April (Figure 3). A total of 33 epiphytic algae species belonging to 6 phyla were identified on *P. crispus* in Liangzi Lake (Supplementary Table S1). Fifteen genera of diatoms, 10 genera of green algae, 6 genera of blue green algae, 1 genus of cryptomonad, 1 genus of euglenoid and 1 genus of golden algae were identified (Supplementary Table S1). Diatoms were the dominant group of epiphytic algae in richness and reached a peak of approximately 10.6 species in mid-March (Figure 4). Green algae had the highest richness in the April with approximately 6.8 species and the lowest richness in December with approximately 1 species (Figure 4). The richness of blue green algae increased over the last three months, reaching a peak of approximately 3 species in mid-June (Figure 4). Only 1 species of euglenoid appeared from March to May (Figure 4). Only 1 species of cryptomonad and golden algae appeared in January and June, respectively (Figure 4).

### Effects of Biotic and Abiotic Environmental Factors on Epiphytic Algal Richness

**FIGURE 5:**
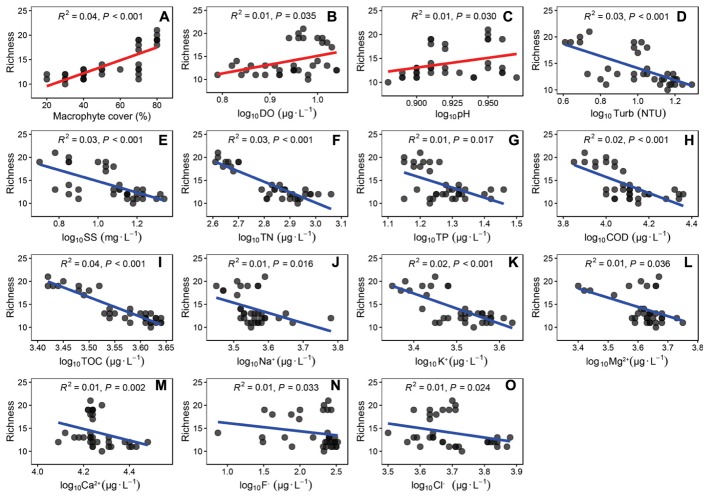
The linear regressions between richness of epiphytic algae and the coverage of *P. crispus* and water quality parameters. Environmental factors were correlated with the richness of epiphytic algae. **(A)** coverage of *P. crispus*, **(B)** dissolved oxygen, **(C)** pH, **(D)** Turbidity, **(E)** total suspended solids, **(F)** Total nitrogen, **(G)** total phosphorus, **(H)** COD, **(I)** total organic carbon, **(J)** Na^+^, **(K)** K^+^, **(L)** Mg^2+^, **(M)** Ca^2+^, **(N)** F^-^ and **(O)** Cl^-^. The regressions coefficients squared and *P*-values are given for the regression by correction for multiple tests (*n* = 35).

The epiphytic algal richness was positively correlated with macrophyte cover, DO and pH (Figures 5A–C). The richness was negatively correlated with Turb, SS, TN, TP, COD, TOC, Na+, K^+^, Ca^2+^, Mg^2+^, F^-^, and Cl^-^ (Figures 5D–O). The epiphytic algal richness was no significantly correlated with T (*R^2^* = 0.00, *P* = 0.176), Cond (*R^2^* = 0.00, *P* = 0.392), Chla (*R^2^* = 0.00, *P* = 0.252) and SO_4_^2-^ (*R^2^* = 0.00, *P* = 0.987).

**FIGURE 6:**
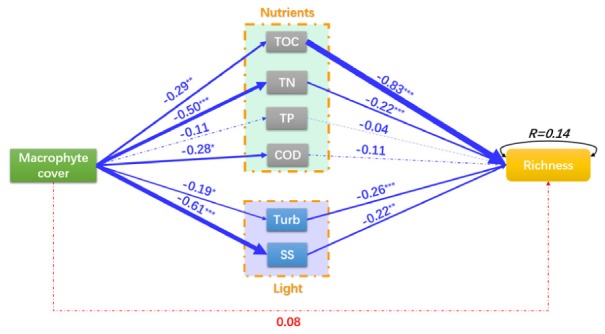
A structural equation model of macrophyte cover effects on the richness of epiphytic algae. Red and blue arrows represent significant positive and negative pathways, respectively. Arrow width is proportional to the strength of the relationship, and solid and dotted lines represent significant and non-significant pathways, respectively. Numbers indicate the standard path coefficients (*C*) *χ^2^*= 242.68, *P* < 0.001; RMSEA = 0.66, *P* < 0.001; AIC = 1216.45. Significance levels are indicated by asterisks: ^∗∗∗^*P* < 0.001, ^∗∗^*P* < 0.01, ^∗^*P* < 0.05.

The macrophyte cover had a significant negative effect on TOC (*C* = –0.29, *P* = 0.003), TN (*C* = –0.50, *P* < 0.001), COD (*C* = –0.28, *P* = 0.003), Turb (*C* = –0.19, *P* = 0.020) and SS (*C* = –0.61, *P* < 0.001) (Figure 6). TOC (*C* = –0.83, *P* < 0.001), TN (*C* = –0.22, *P* < 0.001), Turb (*C* = –0.26, *P* < 0.001), and SS (*C* = –0.22, *P* = 0.001) had a negative effect on the richness of epiphytic algae (Figure 6). The macrophyte cover had a non-significant negative effect on the richness of epiphytic algae (*C* = 0.08, *P* = 0.276, Figure 6). The model shows that *P. crispus* effects the diversity of epiphytic algae by reducing nutrient concentration (TOC and TN decreases) and increasing the clarity of the water (Turb and SS decreases) to improve the richness of epiphytic algae.

## Discussion

Changes in the macrophyte community can be an important cause of changes in the epiphytic algal community structure (Souza et al., 2015). Increasing macrophyte coverage could increase the species richness of epiphytic algae by providing more diverse and heterogeneous habitats for epiphytic algae (Cattaneo et al., 1998; Toporowska et al., 2008; Celewicz-Gołdyn and Kuczyńska-Kippen, 2017). Liangzi Lake had a high abundance of aquatic macrophytes, especially *P. crispus* during winter to early summer, and the abundance varied over this period (Qian et al., 2014). Our results showed that the total richness of epiphytic algae had a similar trend to that of *P. crispus* coverage (Figure 3), which suggested that higher coverage of *P. crispus* might provide more habitats and spatial niches for epiphytic algae. Therefore, within a range of coverage and conditions examined, a greater *P. crispus* coverage could accommodate more species of epiphytic algae.

On the other hand, when the coverage of *P. crispus* increases, total organic carbon, total nitrogen, COD, turbidity and total suspended solids in the water column are decreased. These results may suggest that *P. crispus* improved the water quality at the growing season in terms of improving transparency, decreasing nutrients are represented by the first part of the SEM. A large amount of nutrients and suspended solids in the water column were absorbed for macrophyte growth and reproduction that have been widely confirmed by many studies (Scheffer, 1999; Cao et al., 2018). The water quality was improving, which usually manifested as high transparency, low nutrient concentrations and high biodiversity in a shallow ecosystem (Karr and Dudley, 1981; Gandhi, 2012; Cao et al., 2018). The diversity of epiphytic algae was positively correlated with water with high transparency and few suspended solids (Kollar et al., 2015). Increased radiation and spectrum would support a more heterogeneous environment for the epiphytic algal community that would accommodate more species of epiphytic algae (Algarte et al., 2017). The enhanced transparency improved the richness of epiphytic algae shown on the light pathway in the SEM; thus, the total richness of epiphytic algae increased with the increasing *P. crispus* coverage. Eutrophication has been confirmed as one of the main drivers of biodiversity loss in recent decades (Hillebrand et al., 2007; Isbell et al., 2013; Newbold et al., 2015; Wang et al., 2016). Increasing *P. crispus* coverage was correlated with reduced the nutrients of the water column (*C* = –0.50, *P* < 0.001, TN; *C* = –0.11, *P* = 0.13, TP; *C* = –0.28, *P* = 0.003, COD; *C* = –0.29, *P* = 0.003, TOC) and improved the richness of epiphytic algae, as shown by the nutrient pathway in the SEM. The nutrient increase can lead to cyanobacterial dominance (Dokulil and Teubner, 2000), community structure simplification and biodiversity loss especially in the mesotrophic and eutrophic lakes (Qin et al., 2013). In the decline phase of *P. crispus*, plant decomposition caused nutrients to be released into the water column that led to the overgrowth and dominance of several species epiphytic algae (such as: *G. subclavatum*, *A. exigua*, *C. vulgaris*, *A. flos-aquae* (Lyngb.) and *O. fraca*; Supplementary Table S1); many epiphytic algae were excluded due to the competition for nutrients and space. Moreover, the TOC was the strongest factor effected on the epiphytic algal richness. While, most algae couldn’t utilize organic matter (Lee, 2008), but the bacteria decomposed organic matter into inorganic carbon which could be utilized by epiphytic algae (Jones et al., 2002; Rier and Stevenson, 2002). The increasing inorganic carbon might led to the overgrowth and dominance of several species epiphytic algae which might excluded many epiphytic algae. On the other hand, the effect of organic matters attenuated light in water column (Babin et al., 2003) which might decrease the epiphytic algal richness.

The pathway form the coverage of *P. crispus* to epiphytic algal richness shown a non-significant effects, which indicated that the changes of *P. crispus* coverage cannot direct explain the variation of epiphytic algal richness. The path coefficient which from the coverage of *P. crispus* to epiphytic algal richness via nutrients (*C* = 0.38) was greater than which via light (*C* = 0.18) and direct effect of *P. crispus* coverage (*C* = 0.08). As the result of the above comparison, the indirect effects (adjusted nutrients concentrations and transparency of water column) of the *P. crispus* coverage on epiphytic algal richness was stronger than that direct effect. The SEM clarified the mechanism by which *P. crispus* improved the epiphytic algal richness by absorbing nutrients and increasing the transmittance of water.

We concluded that *P. crispus* affected the richness of epiphytic algae by reducing nutrients concentrations (TOC, TN, and COD decreased) and increasing transparency (Turb and SS decreased). This result suggests that high submerged macrophyte cover can improve the richness of the epiphytic algae community indirectly by changing water qualities.

## Author Contributions

DY and CL designed and executed the research project. TL, QH, and YH collected the data. TL led the reflectance data analysis and drafted the manuscript with the assistance of CL. All the co-authors commented on and approved the final manuscript.

## Conflict of Interest Statement

The authors declare that the research was conducted in the absence of any commercial or financial relationships that could be construed as a potential conflict of interest. The handling Editor is currently organizing a Research Topic with one of the authors CL, and confirms the absence of any other collaboration.
