# Supplementary material for: Effects of Water Quality Adjusted by Submerged Macrophytes on the Richness of the Epiphytic Algal Community
Source: Front Plant Sci. 2019 Jan 9;9:1980. doi: 10.3389/fpls.2018.01980 (PMC6334159; doi:10.3389/fpls.2018.01980)
Supplement: Supplementary file 1 [file Table_1.doc]

**Table S1∣**The abundance of epiphytic algae collected from *P. crispus.* The total abundance of each month was the mean of the 5 fixed sites.

| **Epiphytic algae Taxa** | |  | **Total Abundance (cell/mm^2^)** | | | | | | |
| --- | --- | --- | --- | --- | --- | --- | --- | --- | --- |
|  |  | **ID** | **Dec** | **Jan** | **Feb** | **Mar** | **Apr** | **May** | **Jun** |
| Phylum： | Bacillariophyta |  |  |  |  |  |  |  |  |
| Class： | Centricae |  |  |  |  |  |  |  |  |
| Order： | Coscinodiscales |  |  |  |  |  |  |  |  |
| Family： | Coscinodiscaceae |  |  |  |  |  |  |  |  |
|  | *Cyclotella hubeiana* | BA15 | 126 | 36 | 121 | 99 | 84 | 0 | 0 |
|  | *Melosira granulata* | BA13 | 10 | 65 | 70 | 72 | 139 | 133 | 0 |
| *Melosira granulata var.angutissima* | | BA11 | 189 | 65 | 160 | 55 | 0 | 0 | 0 |
| Class： | Pennatae |  |  |  |  |  |  |  |  |
| Order： | Araphidiales |  |  |  |  |  |  |  |  |
| Family： | Fragilariaceae |  |  |  |  |  |  |  |  |
|  | *Synedra acus* | BA03 | 264 | 51 | 136 | 72 | 139 | 115 | 407 |
|  | *Synedra ulna* | BA07 | 1986 | 2865 | 3695 | 3527 | 1768 | 1334 | 758 |
| Order： | Biraphidinales |  |  |  |  |  |  |  |  |
| Family： | Cymbellaceae |  |  |  |  |  |  |  |  |
|  | *Cymbella aspera* | BA05 | 0 | 0 | 0 | 55 | 139 | 302 | 0 |
| Family： | Gomphonemaceae |  |  |  |  |  |  |  |  |
| *Gomphonema constrictum var.capitatum* | | BA08 | 0 | 0 | 80 | 72 | 1698 | 1433 | 11 |
|  | *Gomphonema gracile* | BA06 | 0 | 92 | 559 | 3527 | 2151 | 1034 | 758 |
|  | *Gomphonema subclavatum* | BA09 | 36 | 65 | 570 | 72 | 1516 | 2551 | 5642 |
| Family： | Naviculaceae |  |  |  |  |  |  |  |  |
|  | *Navicula capitatoradiata* | BA04 | 56 | 0 | 22 | 33 | 0 | 302 | 0 |
|  | *Navicula pupula* | BA21 | 42 | 0 | 0 | 0 | 0 | 0 | 0 |
|  | *Navicula reichardtiana* | BA20 | 126 | 0 | 70 | 34 | 139 | 0 | 0 |
|  | *Stauroneis anceps* | BA12 | 72 | 0 | 0 | 0 | 0 | 0 | 0 |
| Order： | Monoraphidales |  |  |  |  |  |  |  |  |
| Family： | Achnanthaceae |  |  |  |  |  |  |  |  |
|  | *Achnanthes exigua* | BA01 | 80 | 111 | 80 | 137 | 2340 | 2551 | 3016 |
|  | *Cocconeis placentula* | BA10 | 20 | 0 | 0 | 0 | 0 | 0 | 0 |
| Phylum： | Chlorophyta |  |  |  |  |  |  |  |  |
| Class： | Chlorophyceae |  |  |  |  |  |  |  |  |
| Order： | Chlorococcales |  |  |  |  |  |  |  |  |
| Family： | Chlorellaceae |  |  |  |  |  |  |  |  |
|  | *Ankistrodesmus spiralis* | CH02 | 0 | 0 | 0 | 0 | 0 | 0 | 758 |
|  | *Chlorella vulgaris* | CH04 | 300 | 130 | 105 | 134 | 139 | 2280 | 3016 |
|  | *Tetraedron minimum* | CH05 | 0 | 0 | 0 | 55 | 139 | 0 | 0 |
| Family： | Pediastraceae |  |  |  |  |  |  |  |  |
|  | *Pediastrum simples* | CH22 | 0 | 65 | 80 | 0 | 0 | 0 | 0 |
| Family： | Scenedesmaceae |  |  |  |  |  |  |  |  |
|  | *Scenedesmus dimorphus* | CH10 | 0 | 0 | 0 | 55 | 100 | 0 | 0 |
|  | *Scenedesmus quadricauda* | CH07 | 0 | 0 | 0 | 77 | 139 | 2 | 0 |
| Order： | Ulothrichales |  |  |  |  |  |  |  |  |
| Family： | Ulothrichaceae |  |  |  |  |  |  |  |  |
|  | *Planctonema lauterbornii* | CH45 | 0 | 0 | 0 | 55 | 139 | 0 | 0 |
| Order： | Volvocales |  |  |  |  |  |  |  |  |
| Family： | Chlamydomonadaceae |  |  |  |  |  |  |  |  |
|  | *Chlamydomonas globosa* | CH19 | 0 | 0 | 0 | 0 | 139 | 0 | 0 |
| Class： | Zygnematophyceae |  |  |  |  |  |  |  |  |
| Order： | Desmidiales |  |  |  |  |  |  |  |  |
| Family： | Desmidiaceae |  |  |  |  |  |  |  |  |
|  | *Closterium parvlum* | CH18 | 0 | 65 | 0 | 0 | 0 | 0 | 0 |
|  | *Cosmarium obtusatum* | CH28 | 0 | 0 | 0 | 55 | 139 | 0 | 758 |
| Phylum： | Chrysophyta |  |  |  |  |  |  |  |  |
| Class： | Chrysophyceae |  |  |  |  |  |  |  |  |
| Order： | Chromulinales |  |  |  |  |  |  |  |  |
| Family： | Dinobryonaceae |  |  |  |  |  |  |  |  |
|  | *Dinobryon divergens* | CS02 | 0 | 65 | 0 | 0 | 0 | 0 | 0 |
| Phylum： | Cryptophyta |  |  |  |  |  |  |  |  |
| Class： | Cryptophyceae |  |  |  |  |  |  |  |  |
| Order： | Cryptomonadaceae |  |  |  |  |  |  |  |  |
| Family： | Cryptomonadaceae |  |  |  |  |  |  |  |  |
|  | *Cryptomonas erosa* | CR02 | 0 | 0 | 0 | 0 | 0 | 0 | 758 |
| Phylum： | Cyanophyta |  |  |  |  |  |  |  |  |
| Class： | Cyanophyceae |  |  |  |  |  |  |  |  |
| Order： | Chroococcales |  |  |  |  |  |  |  |  |
| Family： | Merismopediaceae |  |  |  |  |  |  |  |  |
|  | *Merismopedia punciata* | CY02 | 0 | 0 | 0 | 0 | 11 | 19 | 0 |
| Order： | Nostocales |  |  |  |  |  |  |  |  |
| Family： | Nostocaceae |  |  |  |  |  |  |  |  |
|  | *Anabaena cylindrica* | CY16 | 0 | 0 | 0 | 0 | 139 | 15 | 0 |
|  | *Anabaena flos-aquae*(Lyngb.) | CY03 | 0 | 0 | 0 | 0 | 0 | 2280 | 3016 |
| Order： | Oscillatoriales |  |  |  |  |  |  |  |  |
| Family： | Oscillatoriaceae |  |  |  |  |  |  |  |  |
|  | *Lyngbya martensiana* | CY13 | 0 | 0 | 0 | 0 | 0 | 17 | 758 |
|  | *Oscillatoria fraca* | CY09 | 134 | 139 | 80 | 137 | 139 | 1433 | 3016 |
| Phylum： | Euglenophyta |  |  |  |  |  |  |  |  |
| Class： | Euglenophyceae |  |  |  |  |  |  |  |  |
| Order： | Euglenales |  |  |  |  |  |  |  |  |
| Family： | Euglenaceae |  |  |  |  |  |  |  |  |
|  | *Euglena sanguinea* | EU01 | 0 | 0 | 0 | 33 | 139 | 1419 | 0 |
